# The visceral and liver fat are significantly associated with the prevalence of hyperuricemia among middle age and elderly people: A cross-sectional study in Chongqing, China

**DOI:** 10.3389/fnut.2022.961792

**Published:** 2022-10-13

**Authors:** Ruixue Bai, Xiuquan Ying, Jieqiang Shen, Tingting Wu, Xingyu Lai, Lingyun Wang, Meng Yu, Xiaoya Qi, Ying Mei

**Affiliations:** ^1^Health Management Center, The Second Affiliated Hospital, Chongqing Medical University, Chongqing, China; ^2^Department of Food and Nutrition, College of Medical and Life Sciences, Silla University, Busan, South Korea

**Keywords:** hyperuricemia, visceral fat area, subcutaneous fat area, liver fat content, middle-age and elderly, China

## Abstract

**Background:**

The prevalence of hyperuricemia (HUA) has been increasing in recent years. HUA is a crucial risk factor for gout and an independent risk factor for cardiovascular diseases (CVDs). Identifying potentially modifiable factors of HUA is vital for preventing gout and even CVDs. This study aimed to explore the associations of fat distribution with HUA among middle-aged and elderly people in Chongqing, China.

**Materials and methods:**

A cross-sectional study was conducted between July 2020 and September 2021. People who underwent quantitative computed tomography (QCT) scans were invited to participate in the study. A total of 3,683 individuals whose clinical characteristics and QCT-based fat distribution measurements included visceral fat area (VFA), subcutaneous fat area (SFA), and liver fat content (LFC) were well-recorded were included. HUA was defined as having a serum uric acid level greater than 420.0 μmol/L. Multivariate logistic regression models were used to evaluate the association between these adipose variables and HUA prevalence.

**Results:**

The HUA prevalence was 25.6% (943/3,683), which was 39.6% (817/2,063) in men and 7.8% (126/1,620) in women. In the fully adjusted model (model 4), the comparison of the highest one with the lowest quartiles of adipose variables showed that the multivariable OR (95% confidence intervals) of HUA were 2.08 (1.36–3.16; *P* for trend = 0.001) for VFA, 0.89 (0.63–1.25; *P* for trend = 0.651) for SFA, and 1.83 (1.42–2.34; *P* for trend < 0.0001) for LFC. For VFA, the association was more evident in men than in women.

**Conclusion:**

Higher VFA and LFC were significantly associated with the increased prevalence of HUA in middle-aged and elderly Chinese individuals. VFA and LFC may have a predictive effect on HUA. Controlling visceral and liver fat accumulation may be beneficial for middle-aged and older people. HUA can be prevented with specific effective healthy physical activity and balanced diet guidelines.

## Introduction

Serum uric acid is the final product of purine metabolism; hyperuricemia (HUA) may develop when the purine metabolism pathway is abnormal (1). Elevated uric acid levels can aggravate diseases with excessive oxidative stress and the inflammatory response (2, 3). HUA is a crucial risk factor for gout and an independent risk factor for cardiovascular, respiratory, and metabolic diseases (4–11). Moreover, elevated serum uric acid is associated with an increased risk of cardiovascular mortality and all-cause mortality (12, 13). According to epidemiological studies, HUA prevalence was 8.5–18.4% from 2001 to 2017 in China; it has become a major public health problem (14). Identifying the potentially modifiable factors of HUA is urgent and essential for the prevention of gout and even other HUA-associated diseases.

Previous studies’ data revealed that being overweight and obese, as well as central obesity, plays a vital role in the management of HUA (15–17). By far, being overweight and obese can be defined by anthropometric measures, including body weight index (BMI) and waist circumference (WC). BMI serves as the “gold standard” for screening obesity. BMI typically represents the total body mass, whereas WC is more reflective of the accumulation of abdominal visceral fat (18). However, BMI and WC cannot accurately identify fat distributions in different regions. A nationwide study in China confirmed that obesity, when defined by traditional anthropometric indices, led to the misclassification of many individuals with increased visceral fat; BMI failed to identify individuals at high risk of having a high proportion of visceral fat (19). In recent years, the relationship between adipose tissue distribution and metabolic diseases has attracted an increasing amount of attention. Many emerging branches of evidence show that visceral adipose tissue can affect local or distant physiological regulation as an endocrine organ with dozens of important active factors, such as leptin and adiponectin, which affect local or distant physiological regulation (20, 21). Abnormal visceral fat distribution is an important risk factor for obesity-related disorders; it is even associated with insulin resistance, lipoprotein metabolism, and hypertension (22, 23). Animal studies have shown that increased xanthine oxidoreductase (XOR) activity in the adipose tissue of obese mice can promote purine catabolism and increase uric acid production (24). We hypothesized that elevated visceral fat is associated with HUA, and the determination of the role of adipose tissue distribution in HUA may be beneficial to HUA prevention in the early phase.

Computed tomography (CT) and magnetic resonance imaging (MRI) are two of the most accurate techniques for measuring the quantity and distribution of adipose tissue. Furthermore, low-dose chest computed tomography (LDCT) scans and abdominal CT scans are frequently used for lung cancer screening and liver, bile, pancreas, spleen, and kidney disease screening in health screenings and checkups. Quantitative CT (QCT) is a quantitative analysis technology based on LDCT or abdominal CT scans that can obtain the abdominal visceral, subcutaneous, and liver fat contents by using the existing CT data without extra radiation (25). Therefore, abdominal adipose tissue measured by QCT is more convenient and time-saving, and extra radiation can be avoided; it has been successfully used in fat distribution studies (26–28). Previously published data on the Japanese population show that the visceral fat area (VFA) and liver fat content (LFC), which are calculated based on abdomen CT scans, are positively associated with HUA (26–28). However, these studies had small sample sizes and participants comprised Japanese men. Moreover, the studies did not consider the constitutive difference in regional body fat distribution between men and women, thereby limiting the robustness and application of the results in other populations. We conducted the first cross-sectional study in middle-aged and elderly Chinese to examine whether visceral, subcutaneous, and liver fat assessed using QCT were independently associated with HUA. Moreover, the potential population with different ages, sex, and clinical characteristics and whose fat distribution presents a positive correlation with HUA prevalence were identified.

## Materials and methods

### Study subjects

All clients who visited the Health Management Center of the Second Affiliated Hospital of Chongqing Medical University for a medical check-up between July 2020 and September 2021 were invited to participate. The clinical significance of visceral and subcutaneous fat tissue accumulation and liver fat content was investigated in 4,688 subjects who underwent a QCT scan. Blood samples were drawn from the antecubital vein of participants after an overnight fast (at least 8 h). Data were extracted from participants who met the following criteria: (a) received a QCT scan; (b) were ≥ 45 years old; and (c) had complete health status indicators. Such indicators included the anthropometric measurements of height, weight, waist circumference (WC), and blood pressure, as well as the biochemical measurement indicators of fasting blood glucose (FBG), blood triglyceride (TG), total cholesterol (TC), high-density lipoprotein cholesterol (HDL-C), and serum uric acid (SUA). Disease history was also considered. A total of 3,683 subjects aged 45–93 years old were finally enrolled. This study was approved by the Ethics Committee of the Second Affiliated Hospital of Chongqing Medical University (approval no. 2020261). Informed consent was obtained from all participants prior to participation.

### Outcome variable: Serum uric acid and hyperuricemia

Serum uric acid was measured by an enzymatic colorimetric method on a Hitachi 7600 automated analyzer (Hitachi Inc., Tokyo, Japan). HUA was defined as having a serum uric acid concentration of ≥ 420 mmol/L in both men and women based on the Guidelines for the Diagnosis and Treatment of HUA and Gout in China (2019) (29).

### Exposure variables: Visceral abdominal fat area, subcutaneous fat area, and liver fat content

Computed tomography was performed using a 64-detector row scanner (Siemens, SOMATOMgo. Top, Germany). The scan parameters were 120 kV, 100 mAs, 1 mm slice thickness, and 40 cm field of view. The QCT calibration phantom (Mindways, Austin, TX, USA) was placed beneath the participants and scanned simultaneously, whereas the CT scanner was working. Details of visceral adipose measurements have been reported previously (25). Total abdominal fat and visceral adipose measurements were performed at the mid-slice of the 2nd lumbar vertebra (L2) by trained and qualified radiologists using Mindways QCT software (QCT-PROV6.1). VFA (cm^2^) was assessed by semiautomated segmentation using the Tissue Composition Module of the Mindways software. The same level of SFA (cm^2^) was calculated by subtracting VFA from the total abdominal fat. LFC was assessed by selecting four circular regions of interest (ROIs) in the liver, with cross-sectional areas of approximately 300 mm^2^. One ROI was selected for the left lobe of the liver, and three ROIs were selected for the right lobe of the liver (30). Finally, the mean value of the four ROIs liver fat was calculated as LFC. The ROIs were placed in the subcapsular region of the liver, avoiding the bile duct and blood vessels. If the left lobe of the liver was too small to be visible in this slice, the slice in which the left lobe had the largest area was used for its measurement. For LFC, the intraclass correlation coefficient (ICC) was 0.96, which indicated good intra-individual consistency for LFC measurements.

### Covariables

Basic information and health status indicators were collected and included as covariates in our analysis. Participants’ ages were classified into two groups, namely, the middle-aged (45–60 years old) and the elderly (≥60 years old). Height (to the nearest 0.1 cm) and weight (to the nearest 0.1 kg) were recorded by the OMRON HNH-219 device by asking participants in lightweight clothing to stand without shoes. BMI was calculated as weight in kilograms divided by height in meters squared and divided into two groups based on the Working Group on Obesity in China (WGOC) criteria recommendation: < 24.0 kg.m^–2^ (non-overweight) and ≥ 24.0 kg.m^–2^ (overweight/obesity) (31). WC was measured at 1 cm above the navel at minimal respiration to the nearest 0.1 cm. Central obesity was defined as a WC of ≥ 90 cm in men and 80 cm in women according to the International Diabetes Federation (IDF) criteria (32). Blood pressure was measured after a 5 min seated test using an electronic sphygmomanometer (OMRON HBP-9020) on the right arm with a selected cuff size based on the upper arm circumference. Hypertension was defined as having a systolic blood pressure (SBP) of ≥ 140 mmHg and/or diastolic blood pressure of ≥ 90 mmHg or having a history of hypertension (33).

Fasting blood glucose was tested by the glucose oxidase-phenol + aminophenazone (GOD-PAP) method on a Bio-Rad D-10 analyzer. Diabetes was defined as having an FBG of ≥ 7.0 mmol/L or having a history of diabetes (34). Serum lipids, including TG, TC, and HDL-C assays, were performed on an automated biochemical analyzer (HITACHI 7600 Series, Chengdu, China). TG was assayed by glycerol-3-phosphate oxidase-phenol + aminophenazone (GPO-PAP) methods. TC was assayed by the cholesterol oxidase-phenol + aminophenazone (CHOD-PAP) method, and HDL-C was assayed. Hyperlipidemia was defined as TC ≥ 6.2 mmol/L, TG ≥ 2.3 mmol/L, or HDL-C < 1.0 mmol/L based on Chinese guidelines for the prevention and treatment of dyslipidemia in adults (35).

### Statistical analysis

Descriptive statistics were used to show the basic information of participants with different serum uric acid statuses. Data were expressed as the means and standard deviations (mean ± SD) for normally distributed continuous variables or the median (25th–75th percentile) for non-normally distributed continuous variables and categorical variables using their frequencies and percentages [n (%)]. Differences in mean and median values were evaluated by using Student’s *t*-test and the Mann–Whitney *U* test, respectively. The chi-square test was used for categorical variables. Multiple logistic regression analysis was used to estimate the odds ratio for the prevalence of hyperuricemia. Four models were developed. Model 1 was adjusted for sex and age (middle-aged or elderly). Model 2 was further adjusted for hypertension (yes or no), hyperlipidemia (yes or no), and diabetes (yes or no). Model 3 was additionally adjusted for the BMI group (non-overweight and overweight/obese) and central obesity (yes or no). Model 4 was additionally adjusted for each other [adipose tissue variables (continuous: cm^2^ or %)]. The results are reported for the odds ratio (OR) and 95% confidence interval (95% CI).

We stratified our analyses by baseline sex, age, BMI group, central obesity, hypertension, hyperlipidemia, and diabetes. *P* for trend was calculated across quartiles by using a multivariable logistic regression model and adjusted for sex, age, BMI, hypertension, diabetes, hyperlipidemia, central obesity, and other adipose variables. The interaction was examined by the log-likelihood ratio test, entering a cross-product term of adipose variables and the stratification variables as ordinal variables. A *P*-value < 0.05 was considered statistically significant. All analyses were performed using Stata version 17.1 software (Stata Corporation, College Station, TX, USA).

## Results

### Characteristics of participants by serum uric acid

Study participants had a median age of 54 years old (ranging from 45 years old to 93 years old). Overall, the prevalence of HUA was 25.6% (943/3,683), which was respectively 39.6% (817/2,063) in men and 7.8% (126/1,620) in women. The characteristics of the participants by serum uric acid status (non-HUA and HUA) are shown in Table 1. Participants with HUA were more likely to be men and more likely to have higher BMI, WC, SBP, DBP, FBG, TG, and LDL-C and greater VFA and LFC. The proportion of overweight/obesity, central obesity, and hypertension was higher in participants with HUA than in participants without HUA (*p* < 0.05). However, the mean levels of HDL-C and SFA were lower in participants who had HUA than in those who did not (*p* < 0.05).

**TABLE 1:** Characteristics of the participants according to serum uric acid status (*N* = 3,683).

Characteristic	Total	Non-hyperuricemia	Hyperuricemia	*P*-value
Patients (n)	3,683	2,740	943	–
Age (years)	54 (50–59)	54 (50–59)	54 (50–58)	0.372
Age group (n[%])				0.22
Middle (45 ≤ Age[y] < 60)	2,838 (77.1)	2,096 (76.5)	742 (78.7)	
Elderly (Age[y] ≥ 60)	845 (22.9)	644 (23.5)	201 (21.3)	
Gender (n[%])				<0.0001
Women	1,620 (43.9)	1,494 (92.2)	126 (7.8)	
Men	2,063 (56.1)	1,246 (60.4)	817 (39.6)	
BMI (kg/m2)	24.1 (22.3–26.1)	23.6 (21.9–25.6)	25.3 (23.6–27.2)	<0.0001
WC (cm)	82 (76–89)	80 (74–87)	88 (82–93)	<0.0001
SBP (mmHg)	125 (114–138)	124 (113–137)	128 (117–140)	<0.0001
DBP (mmHg)	75 (68–83)	74 (67–82)	78 (71–86)	<0.0001
FBG (mmol/L)	5.1 (4.7–5.6)	5.0 (4.6–5.5)	5.1 (4.7–5.7)	0.0004
TC (mmol/L)	5.2 (4.6–5.9)	5.2 (4.6–5.9)	5.3 (4.6–5.9)	0.646
TG (mmol/L)	1.5 (1.1–2.2)	1.4 (1.0–1.9)	2.0 (1.4–2.9)	<0.0001
HDL-C (mmol/L)	1.3 (1.1–1.5)	1.3 (1.1–1.6)	1.2 (1.0–1.3)	<0.0001
LDL-C (mmol/L)	2.9 (2.4–3.4)	2.9 (2.4–3.4)	2.9 (2.4–3.5)	0.0005
SUA (μmol/L)	357.1 (297.8–421.7)	326.7 (281.1–370.6)	471.8 (443.2–515.8)	<0.0001
BMI group (n[%])				<0.0001
Non-overweight	1,787 (48.5)	1,512 (55.2)	275 (29.2)	
Overweight/obesity	1,896 (51.5)	1,228 (44.8)	668 (70.8)	
Central obesity (n[%])	1,301 (35.2)	849 (30.9)	452 (47.7)	<0.0001
Hypertension (n[%])	1,155 (31.4)	796 (29.0)	359 (38.1)	<0.0001
Diabetes (n[%])	386 (10.5)	289 (10.5)	97 (10.3)	0.806
Hyperlipidemia (n[%])	1,590 (43.2)	1,031 (37.6)	559 (59.3)	<0.0001
VFA (cm^2^)	156.6 (108.3–212.1)	138.5 (97.4–192.1)	206.3 (164.4–252.2)	<0.0001
SFA (cm^2^)	96.3 (73.1–127.1)	98.8 (73.5–132.1)	90.7 (72.5–113.7)	<0.0001
LFC (%)	6.0 (3.8–9.1)	5.6 (3.6–8.3)	7.7 (4.7–11.5)	<0.0001

BMI, body mass index; WC, Waist circumference; SBP, systolic blood pressure; DBP, diastolic blood pressure; FPG, fasting plasma glucose; TC, total cholesterol; TG, triglycerides; HDL-C, high-density lipoprotein cholesterol; LDL-C, low-density lipoprotein cholesterol; SUA, serum uric acid; VFA, visceral abdominal fat area; SFA, subcutaneous fat area; LFC, liver fat content; WC, waist circumference.

Data were expressed as means and standard deviations (mean ± SD) for normally distributed continuous variables, or the median (25th–75th percentile) for non-normally distributed continuous variables, and categorical variables using their frequencies and percentages[n (%)].

**FIGURE 1:**
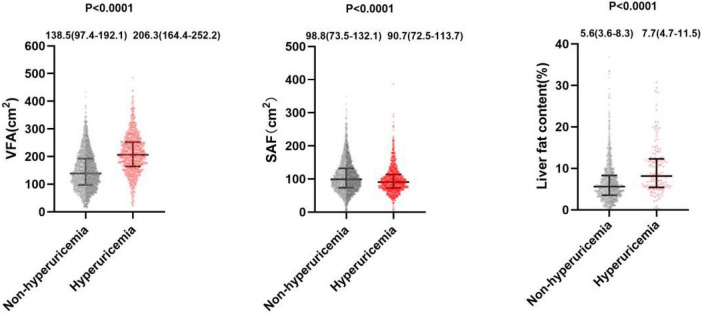
Relationship between hyperuricemia and VFA, SAF, or LFC. VFA and LFC are significantly higher in patients with HUA than in those without (VFA: 206.3 [164.4–252.2]cm^2^, *n* = 2,749 vs. 138.5 [97.4–192.1]cm^2^, *n* = 948, *P* < 0.0001; LFC: 7.7 [4.7–11.5]%, *n* = 2,749 vs. 5.6 [3.6–8.3]%, *n* = 948, *P* < 0.0001), whereas SFA is significantly lower in patients with HUA than in those without (90.7 [72.5–113.7]cm^2^, *n* = 2,749 vs. 98.8 [73.5–132.1]cm^2^, *n* = 948, *P* < 0.0001).

As shown in Figure 1, participants with HUA exhibited significantly higher VFA and liver fat content than those without HUA [VFA: 206.3 (164.4–252.2) cm^2^, *n* = 943 vs. 138.5 (97.4–192.1) cm^2^, *n* = 2,740, *p* < 0.0001; LFC: 7.7 (4.7–11.5)%, *n* = 943 vs. 5.6 (3.6–8.3)%, *n* = 2,740, *p* < 0.0001], whereas there was a significantly lower SFA in HUA participants than in non-HUA participants [90.7 (72.5–113.7) cm^2^, *n* = 943 vs. 98.8 (73.5–132.1) cm^2^, *n* = 2,740, *p* < 0.0001].

### Association between adipose variables and hyperuricemia prevalence (*N* = 3,683)

A multivariate logistic regression analysis was used to examine the association between adipose variables (VFA, SFA, and LFC) and HUA Prevalence (Table 2). In a logistic regression analysis of VFA and HUA, all four models showed a positive association between the VFA and HUA prevalence in the highest VFA group. In the fully adjusted model (model 4), across quartiles of VFA, the OR (95% CI) for prevalent HUA were 1, 1.53 (1.08–2.17), 1.90 (1.31–2.76), and 2.08 (1.36–3.16), respectively (*P* for trend < 0.0001). For the same comparison, we observed a significant positive association between LFC and HUA prevalence. The OR (95% CI) for HUA prevalence across quartiles of LFC was 1, 1.12 (0.87–1.44), 1.33 (31.04–1.71), and 1.83 (1.42–2.34) (*P* for trend < 0.0001).

**TABLE 2:** Association between adipose variables and hyperuricemia prevalence (*N* = 3,683).

	Quartiles of adipose variables				*P* for trend*
	Quartile 1	Quartile 2	Quartile 3	Quartile 4	
VFA					
Model 1^a^	1.00 (ref)	2.01 (1.45–2.79)	3.33 (2.42–4.59)	5.08 (3.67–7.03)	<0.0001
Model 2^b^	1.00 (ref)	1.86 (1.34–2.59)	2.86 (2.06–3.97)	4.04 (2.88–5.67)	<0.0001
Model 3^c^	1.00 (ref)	1.63 (1.16–2.27)	2.12 (1.49–3.02)	2.43 (1.63–3.62)	<0.0001
Model 4^d^	1.00 (ref)	1.53 (1.08–2.17)	1.90 (1.31–2.76)	2.08 (1.36–3.16)	0.001
SFA					
Model 1^a^	1.00 (ref)	1.57 (1.27–1.94)	1.89 (1.51–2.36)	2.34 (1.79–3.06)	<0.0001
Model 2^b^	1.00 (ref)	1.43 (1.15–1.77)	1.68 (1.34–2.12)	1.97 (1.49–2.59)	<0.0001
Model 3^c^	1.00 (ref)	1.13 (0.90–1.42)	1.13 (0.88–1.46)	1.00 (0.72–1.41)	0.734
Model 4^d^	1.00 (ref)	0.99 (0.79–1.26)	1.01 (0.78–1.31)	0.89 (0.63–1.25)	0.651
LFC					
Model 1^a^	1.00 (ref)	1.19 (0.93–1.53)	1.57 (1.24–1.99)	2.78 (2.21–3.50)	<0.0001
Model 2^b^	1.00 (ref)	1.20 (0.93–1.54)	1.52 (1.19–1.93)	2.43 (1.92–3.08)	<0.0001
Model 3^c^	1.00 (ref)	1.14 (0.88–1.46)	1.37 (1.07–1.75)	1.97 (1.54–2.51)	<0.0001
Model 4^d^	1.00 (ref)	1.12 (0.87–1.44)	1.33 (31.04–1.71)	1.83 (1.42–2.34)	<0.0001

VFA, visceral abdominal fat area; SFA, subcutaneous fat area; LFC, liver fat content. All values OR (95% CI).

*P for trend was calculated across quartiles using multivariable logistic regression models.

^*a*^Model1 adjusted for age and gender.

^*b*^Model2 further adjusted for hypertension, hyperlipidemia, and diabetes.

^*c*^Model3 further adjusted for BMI and WC.

^*d*^Model4 further adjusted for other adipose variables (log transferred).

In models 1 and 2, higher quintiles of SFA were significantly associated with increased odds of HUA compared with the lowest quintile. However, the association was attenuated and became non-significant after further adjustment for BMI and WC (model 3) (*P* for interaction = 0.734). Similar results were found in the fully adjusted model (model 4) by further adjusting log VFA and log LFC (*P* for trend = 0.651).

### Stratified associations between adipose variables and hyperuricemia by sex, age, body weight index, hypertension, diabetes, hyperlipidemia, and central obesity (*N* = 3,683)

In stratified analyses, no interactions were observed between the adipose variables (VFA, SFA, and LFC) and sex, age, BMI, hypertension, diabetes, hyperlipidemia, and central obesity for the HUA odds (Table 3). In the subgroups of men, the OR (95% CI) for prevalent HUA was 2.68 (95% CI 1.53–4.70) in the group with the highest VFA in comparison with the group with the lowest.

**TABLE 3:** Stratified associations between VFA, SFA, LFC, and hyperuricemia by sex, age, BMI, hypertension, diabetes, hyperlipidemia, and central obesity (*N* = 3,683).

	Quartiles of adipose variables				*P* for trend*	*P* for interaction†
	Quartile 1 (low)	Quartile 2	Quartile 3	Quartile 4 (high)		
**VFA (cm^2^)**						
Sex						
Women (*n* = 1,620)	1.00 (ref)	0.94 (0.55–1.58)	1.25 (0.67–2.35)	0.70 (0.26–1.94)	0.916	0.38
Men (*n* = 2,063)	1.00 (ref)	1.87 (1.09–3.20)	2.21 (1.30–3.78)	2.68 (1.53–4.70)	0.001	
**Age (y)**						
Middle (*n* = 2,838)	1.00 (ref)	1.44 (0.96–2.16)	1.73 (1.12–2.67)	1.86 (1.15–3.02)	0.02	0.456
Elderly (*n* = 845)	1.00 (ref)	1.57 (0.77–3.21)	2.23 (1.05–4.74)	2.45 (1.03–5.83)	0.043	
**BMI group**						
Non-overweight (*n* = 1,787)	1.00 (ref)	1.67 (1.08–2.58)	2.08 (1.27–3.40)	1.85 (0.98–3.50)	0.024	0.388
Overweight/obesity (*n* = 1,896)	1.00 (ref)	1.02 (0.52–1.99)	1.36 (0.70–2.62)	1.58 (0.79–3.15)	0.038	
**Hypertension**						
No (*n* = 2,528)	1.00 (ref)	1.59 (1.06–2.38)	2.06 (1.33–3.20)	1.95 (1.17–3.23)	0.02	0.487
Yes (*n* = 1,155)	1.00 (ref)	1.32 (0.64–2.71)	1.57 (0.75–3.25)	2.10 (0.96–4.63)	0.029	
**Diabetes**						
No (*n* = 3,297)	1.00 (ref)	1.52 (1.06–2.19)	1.97 (1.34–2.91)	2.02 (1.30–3.13)	0.004	0.26
Yes (*n* = 386)	1.00 (ref)	1.17 (0.28–4.83)	1.06 (0.25–4.45)	1.75 (0.38–8.00)	0.253	
**Hyperlipidemia**						
No (*n* = 2,093)	1.00 (ref)	1.30 (0.82–2.06)	1.64 (1.00–2.71)	1.73 (0.97–3.06)	0.054	0.867
Yes (*n* = 1,590)	1.00 (ref)	1.80 (1.04–3.13)	2.30 (1.29–4.11)	2.57 (1.36–4.88)	0.01	
**Central obesity**						
No (*n* = 2,385)	1.00 (ref)	1.40 (0.93–2.11)	1.88 (1.21–2.94)	1.79 (1.08–2.97)	0.021	0.171
Yes (*n* = 1,298)	1.00 (ref)	1.11 (0.50–2.48)	1.21 (0.55–2.66)	1.75 (0.77–3.98)	0.043	
**SFA (cm^2^)**						
**Sex**						
Women (*n* = 1,620)	1.00 (ref)	0.77 (0.27–2.23)	0.85 (0.28–2.59)	0.91 (0.23–3.63)	0.907	0.178
Men (*n* = 2,063)	1.00 (ref)	0.97 (0.72–1.30)	0.92 (0.62–1.36)	0.57 (0.33–0.99)	0.105	
**Age (y)**						
Middle (*n* = 2,838)	1.00 (ref)	0.96 (0.70–1.31)	1.73 (1.12–2.67)	1.86 (1.15–3.02)	0.076	0.259
Elderly (*n* = 845)	1.00 (ref)	1.57 (0.77–3.21)	2.23 (1.05–4.74)	2.45 (1.03–5.83)	0.776	
**BMI group**						
Non-overweight (*n* = 1,787)	1.00 (ref)	0.95 (0.27–1.35)	1.13 (0.70–1.83)	1.07 (0.52–2.20)	0.761	0.912
Overweight/obesity (*n* = 1,896)	1.00 (ref)	1.11 (0.80–1.54)	1.07 (0.76–1.50)	0.83 (0.56–1.24)	0.369	
**Hypertension**						
No (*n* = 2,528)	1.00 (ref)	1.59 (1.06–2.38)	2.06 (1.33–3.20)	1.95 (1.17–3.23)	0.155	0.659
Yes (*n* = 1,155)	1.00 (ref)	1.32 (0.64–2.71)	1.57 (0.75–3.25)	2.10 (0.96–4.63)	0.478	
**Diabetes**						
No (*n* = 3,297)	1.00 (ref)	0.96 (0.72–1.29)	0.86 (0.59–1.25)	0.65 (0.39–1.11)	0.122	0.279
Yes (*n* = 386)	1.00 (ref)	0.71 (0.31–1.61)	0.89 (0.32–2.45)	0.67 (0.16–2.75)	0.759	
**Hyperlipidemia**						
No (*n* = 2,093)	1.00 (ref)	1.30 (0.82–2.06)	1.64 (1.00–2.71)	1.73 (0.97–3.06)	0.411	0.139
Yes (*n* = 1,590)	1.00 (ref)	1.80 (1.04–3.13)	2.30 (1.29–4.11)	2.57 (1.36–4.88)	0.152	
**Central obesity**						
No (*n* = 2,385)	1.00 (ref)	1.40 (0.93–2.11)	1.88 (1.21–2.94)	1.79 (1.08–2.97)	0.403	0.669
Yes (*n* = 1,298)	1.00 (ref)	1.11 (0.50–2.48)	1.21 (0.55–2.66)	1.75 (0.77–3.98)	0.85	
**LFC (%)**						
**Sex**						
Women (*n* = 1,620)	1.00 (ref)	1.91 (1.01–3.61)	1.88 (0.98–3.61)	3.02 (1.63–5.61)	0.001	0.064
Men (*n* = 2,063)	1.00 (ref)	1.01 (0.76–1.34)	1.26 (0.96–1.66)	1.61 (1.22–2.12)	<0.0001	
**Age (y)**						
Middle (*n* = 2,838)	1.00 (ref)	1.11 (0.83–1.48)	1.38 (1.04–1.83)	1.83 (1.38–2.43)	<0.0001	0.731
Elderly (*n* = 845)	1.00 (ref)	1.09 (0.63–1.89)	1.16 (0.68–1.98)	1.57 (0.92–2.67)	0.078	
**BMI group**						
Non-overweight (*n* = 1,787)	1.00 (ref)	1.48 (1.02–2.15)	1.51 (1.02–2.22)	2.17 (1.41–3.33)	0.001	0.281
Overweight/obesity (*n* = 1,896)	1.00 (ref)	0.89 (0.63–1.26)	1.19 (0.86–1.64)	1.56 (1.14–2.13)	<0.0001	
**Hypertension**						
No (*n* = 2,528)	1.00 (ref)	1.13 (0.84–1.53)	1.31 (0.97–1.78)	1.81 (1.33–2.46)	<0.0001	0.987
Yes (*n* = 1,155)	1.00 (ref)	1.07 (0.66–1.74)	1.35 (0.87–2.09)	1.72 (1.12–2.64)	0.005	
**Diabetes**						
No (*n* = 3,297)	1.00 (ref)	1.17 (0.90–1.52)	1.33 (1.03–1.72)	1.82 (1.40–2.36)	<0.0001	0.536
Yes (*n* = 386)	1.00 (ref)	0.58 (0.20–1.68)	1.17 (0.48–2.88)	1.40 (0.58–3.35)	0.106	
**Hyperlipidemia**						
No (*n* = 2,093)	1.00 (ref)	1.04 (0.74–1.48)	1.47 (1.05–2.07)	1.98 (1.37–2.86)	<0.0001	0.563
Yes (*n* = 1,590)	1.00 (ref)	1.25 (0.86–1.82)	1.22 (0.85–1.76)	1.69 (1.20–2.38)	0.003	
**Central obesity**						
No (*n* = 2,385)	1.00 (ref)	1.30 (0.96–1.77)	1.39 (1.02–1.89)	2.00 (1.44–2.79)	<0.0001	0.37
Yes (*n* = 1,298)	1.00 (ref)	0.81 (0.51–1.29)	1.15 (0.75–1.78)	1.46 (0.98–2.18)	0.006	

BMI, body mass index; VFA, visceral abdominal fat area; SFA, subcutaneous fat area; LFC, liver fat content.

*P trend calculated across quartiles using multivariable logistic regression model. Adjusted for sex, age group, BMI group, hypertension, diabetes, hyperlipidemia, central obesity, and each other adipose variables (log transferred).

^†^Calculated using likelihood ratio test.

The associations among VFA, LFC, and increasing HUA prevalence were only significant among participants with no diabetes compared with those with diabetes. A significant positive relationship between the LFC and HUA was found in the middle-aged (45–65 years old) participants (OR: 1.83, 95% CI 1.38–2.43), but not in the participants aged 65 years old and older.

## Discussion

Many epidemiological studies revealed that the morbidity of HUA is ever growing and accompanied by metabolic syndrome (36–39). However, there is no cost-effective strategy for preventing the occurrence of HUA. Here, we performed the first cross-sectional study on this topic and demonstrated that higher VFA and LFC present a positive association with prevalent HUA in middle-aged and older Chinese populations. This finding indicated that controlling VFA and LFC may effectively prevent HUA occurrence and development. We further identified the potential population along with different ages, sex, and clinical characteristics; the fat distribution of such a population shows a positive correlation with HUA prevalence. The population displayed predisposition as follows. (i) The associations between higher VFA and prevalent HUA were significant in men and people with no diabetes compared with women and people with diabetes, respectively. (ii) The associations between higher LFC and prevalent HUA were significant in people with no diabetes and the middle-aged (45–65 years old) participants compared with people with diabetes and participants aged 65 years old and older, respectively.

Hyperuricemia is caused by reduced renal/external excretion and overproduction of uric acid. It increased the risk of major cardiovascular events and decreased the overall quality of life. Identifying risk factors for HUA is conducive to preventing its occurrence and progression. The present findings on the positive association between the accumulation of visceral fat and HUA are consistent with those obtained in previous studies on other races and ethnicities. A cross-sectional study performed on 801 Japanese individuals aged 25–77 years old demonstrated that visceral and liver fat are positively associated with HUA in multiple logistic regression analysis (26). Hikita et al. studied 508 Japanese men aged 24–68 years old, and Takahashi et al. studied 50 Japanese men aged 29–78 years and reported that higher levels of VFA were associated with elevated concentrations of serum uric acid in multiple linear regression analysis, but there was no significant correlation between SFA and serum uric acid levels in all these studies (27, 28). These observations suggested that higher VFA and LFC present a positive association with HUA prevalence among men located in East Asia. It may be a cost-effective way to prevent HUA through the decline in VFA and LFC for East Asian men. However, previous studies did not present the same observation on women and did not explore whether the fat distribution has a positive correlation with the prevalence of HUA. In this study, VFA presented a positive correlation with HUA prevalence in men, but not in women, which suggested that the effect of VFA on HUA varied in different populations. The stores of surplus calories are different between men and women. Men are much more likely to store fat as visceral fat whereas women are more likely to store it as subcutaneous fat due to different genetic backgrounds, and hormonal differences (40). Interestingly, participants with no diabetes were more likely to have HUA when they had high VFA and LFC. Diabetes regularly requires the administration of hypoglycemic drugs to maintain appropriate blood glucose levels and to reduce complications. Data from *in vitro* and *in vivo* research showed that hypoglycemic drugs can decrease the concentration of serum uric acid (41). Further study is needed to determine the exact mechanisms of these observations.

The mechanism of HUA derived from VFA and LFC has not been elucidated. The causes of HUA are the overproduction of uric acid, the reduction in renal or external excretion of uric acid, or a combination of the two (42). Gene expression detection and enzyme-linked immunosorbent assay showed that the expression and activity of xanthine oxidoreductase (XOR) were higher during visceral and liver fat accumulation. XOR is a rate-limiting enzyme in the liver that catalyzes the conversion of hypoxanthine to xanthine, which is the precursor of uric acid. A previous study indicated that XOR is a mediator of the relationship between non-alcoholic fatty liver disease (NAFLD) and HUA, which indicated that XOR is an important regulatory factor in visceral and liver fat accumulation and uric acid production (43, 44). Moreover, both visceral and liver fat are likely to result in insulin resistance and hyperinsulinemia, which may affect renal excretion and reabsorption of serum uric acid, resulting in reduced excretion of serum uric acid to elevate serum uric acid (45–48).

For most patients with HUA, preventing medication and controlling seafood intake and alcohol consumption are the main treatment methods. Higher VFA and LFC present a positive association with a higher prevalence of HUA. To prevent visceral or liver fat accumulation, physical activity is a critical target that is inversely related to HUA prevalence, as shown by epidemiological data (49–52). These results indicated that specific operational guidelines for physical activity and a balanced diet for patients with HUA, especially middle-aged and older Chinese populations, should be constructed and applied in the future. These may satisfactorily prevent HUA. There are no standard cutoff values of visceral and liver fat among middle-aged and elderly people worldwide, and developing standard cutoff values of visceral and liver fat in further studies is needed to prevent and control HUA among middle-aged and elderly people.

This study had certain limitations. First, it was a cross-sectional study. The relation between adipose variables and HUA was only observed at one point in time. Thus, we could not establish causal relationships. Longitudinal studies are necessary to examine the causal role of adipose variables and HUA. Second, the current study did not obtain diet or physical activity lifestyle factors, which may affect the serum uric acid concentration of participants. Further research on these topics is necessary. Third, we only collected data in this regional medical center. Further study in a multicenter setting is needed to improve the study design.

## Conclusion

This is the first study to demonstrate that both visceral and liver fat (scanned by QCT) were associated with an increased prevalence of HUA in middle-aged and older Chinese individuals, whereas the associations were not significant in subcutaneous fat. Visceral fat or liver fat may have a predictive effect on HUA prevention. Specific effective healthy physical activity and balanced diet guidelines for middle-aged and older people who have specific physical activity need to be developed for HUA prevention. These would control visceral and liver fat accumulation for the specific population. Standard cutoff values of visceral and liver fat in further studies need to be developed to prevent and control HUA among middle-aged and elderly people.

## Data availability statement

The raw data supporting the conclusions of this article will be made available by the authors, without undue reservation.

## Ethics statement

This study was approved by the Ethics Committee of The Second Affiliated Hospital of Chongqing Medical University (approval no. 2020261). The patients/participants provided their written informed consent to participate in this study.

## Author contributions

XQ and YM conceived and designed the study and helped to perform the study. RB, XY, JS, XL, LW, and MY performed the study. RB analyzed the data and wrote the manuscript. TW helped with data interpretation. All of the authors helped to draft the manuscript and read and approved the final manuscript.
